# Importance of Electrodiagnostic Testing Prior to a Tethered Cord Release in a Patient with Overlapping Symptoms of Charcot-Marie-Tooth Disease

**DOI:** 10.7759/cureus.32076

**Published:** 2022-11-30

**Authors:** Lisa B Shields, Vasudeva G Iyer, Yi Ping Zhang, Christopher B Shields

**Affiliations:** 1 Neurological Surgery, Norton Neuroscience Institute, Norton Healthcare, Louisville, USA; 2 Neurology, Neurodiagnostic Center of Louisville, Louisville, USA

**Keywords:** radiology, neurosurgery, genetics, ultrasound, nerve conduction studies, electromyography, tethered cord release, tethered cord syndrome, charcot-marie-tooth disease, neurology

## Abstract

Charcot-Marie-Tooth (CMT) disease is the most common inherited neuromuscular disorder causing a symmetric, slowly progressive distal neuropathy of the legs and arms. Tethered cord syndrome (TCS) encompasses a constellation of neurological, gastrointestinal, musculoskeletal, and urinary abnormalities resulting from spinal cord traction. The signs and symptoms of CMT and TCS may be very similar. Electrodiagnostic (EDX) studies are crucial in differentiating between these two conditions. We describe a 20-year-old woman with a history of low back pain, right leg tingling, and difficulty in walking. She experienced bowel and urinary incontinence 11 years earlier as well as back pain, in-toeing gait, and difficulties with balance and coordination. Urodynamic studies and cervical, thoracic, and lumbar magnetic resonance imaging (MRI) were normal, with the latter demonstrating the tip of the conus medullaris in the normal position at the L1-2 interspace and the filum terminale demonstrating no thickening or fatty infiltration. Despite the absence of radiological evidence of TCS, she underwent a tethered cord release (TCR) with improvement of the pain postoperatively. Two years later, the back pain recurred with increasing pain, paresthesia, and weakness of the lower extremities with frequent trip and fall episodes. EDX studies of the lower extremities were requested. Clinical findings included weakness of flexion and extension of the toes as well as dorsiflexors of the ankles bilaterally. The knee and ankle reflexes were absent bilaterally. EDX testing of the lower extremities were non-diagnostic due to loss of sensory and compound muscle action potentials. EDX studies of the upper extremities suggested a demyelinating polyneuropathy, most likely CMT disease. Genetic studies subsequently confirmed CMT type 1A (CMT1A) disease. Ensuing EDX studies on the patient’s father and brother also suggested CMT1A disease. Physicians should be aware of the overlapping signs and symptoms of CMT disease and TCS. Performing comprehensive EDX studies prior to a TCR particularly in cases when a lumbar MRI is negative may prove valuable in identifying cases of CMT disease with subsequent confirmation by genetic testing.

## Introduction

Charcot-Marie-Tooth (CMT) disease, the most frequent hereditary neuromuscular disorder affecting one in 2500 individuals, is a heterogeneous group of motor-sensory length-dependent neuropathies affecting either the myelin and/or axons of peripheral nerves [1,2]. Inherited in an autosomal dominant manner, CMT type 1A (CMT1A) disease is the most common form of CMT disease and is caused by a 1.5 Mb duplication on chromosome 17p11.2 encompassing the gene coding for peripheral myelin protein-22 (PMP22 gene) [1-4]. This condition is characterized by distal muscle weakness, sensory loss, decreased deep tendon reflexes (DTRs), and skeletal deformities such as pes cavus [1,3,5]. Common electrodiagnostic (EDX) findings in demyelinating CMT1A disease include diffuse and homogeneous slowing of nerve conduction velocities (NCVs) (ulnar and median motor NCV ≤ 38 m/s) without a conduction block [2-4]. A nerve biopsy often reveals well-formed onion-bulb formations seen on histopathology. A high-resolution ultrasound (US) shows diffuse enlargement of nerves as evidenced by an increase in the cross-sectional area (CSA) over the length of the nerve [2].

With an incidence of 0.05-0.25 per 1,000 births, tethered cord syndrome (TCS) may involve neurological, gastrointestinal, musculoskeletal, and urinary dysfunction that is due to spinal cord traction and loss of elasticity of the filum terminale (FT) [6-11]. Traction on the caudal spinal cord results in diminished blood flow, leading to metabolic derangements responsible for sensorimotor and urinary dysfunction [7]. TCS is a subcategory of occult spinal dysraphism, marked by cutaneous stigmata (nevi, lipomas, hair tufts, dermal sinuses, and hemangiomas), progressive lower extremity orthopedic deformities, bowel and bladder deficits (including neurogenic bladder with repeated urinary tract infections and urinary incontinence), progressive sensorimotor deficits, gait abnormalities (toe-walking and clumsiness), easy leg fatiguability, and scoliosis [6,8,10,11]. While TCS may present at any age based on the underlying pathologic condition, it is most commonly diagnosed in infants and children [6,10]. Patients in their late childhood/teenage years may present with nondermatomal pain in the lumbosacral region, perineum, and legs in conjunction with sphincter dysfunction and incontinence [8]. Distinctive findings on lumbar magnetic resonance imaging (MRI) include a low-lying conus medullaris (CM) (below the L1-2 disc space) and thickened FT (diameter > 2 mm) [7-9]. Urodynamic studies (UDS) are the gold standard for urological assessment in patients with suspected TCS [11]. This procedure evaluates lower urinary tract function and often reveals detrusor hyperreflexia in TCS. A tethered cord release (TCR) involves surgical detethering via a one-level lumbar laminectomy which restores blood flow to the lower spinal cord and CM and resolves pain in most cases [6,7,10]. 

This case report illustrates the overlapping signs and symptoms of CMT1A disease and TCS. The importance of EDX and US studies in differentiating these two conditions is discussed. 

## Case presentation

A 20-year-old woman (BMI: 31.83 kg/m^2^) referred for EDX studies reported the spontaneous onset of increased low back pain as well as pain and tingling of the lateral aspect of the right thigh, calf, and sole of the foot. She had difficulty walking with a tendency to trip and fall. She used ankle braces. 

At nine years of age, the patient had experienced a two-month duration of bowel incontinence followed by urinary incontinence one year later. The bowel incontinence resolved, while the nocturnal enuresis persisted. Her right leg was shorter than the left, the right calf was 2.5 cm less than the left in diameter, and the patient displayed in-toeing gait. Over the next two years, she continued to experience increasing back pain, deteriorating gait, and difficulties with balance and coordination. The patient also had worsening of tightness and inward turning of her right leg, to the extent that she sustained a fracture of her right foot. Cervical, thoracic, and lumbar MRIs without gadolinium contrast were normal, with the latter imaging study demonstrating the tip of the CM in the normal position at the L1-2 interspace and the FT demonstrating no thickening or fatty infiltration. These findings revealed no evidence of a tethered cord. UDS and a renal sonogram were normal. At 11 years of age, a TCR was performed for “questionable tethered cord” and “several signs and symptoms potentially consistent with tethering”. Although the patient attained improvement in her low back pain postoperatively within two years, she experienced a recurrence of back pain with a radiating component to the legs as well as muscle spasms. The patient was treated with lioresal 5 mg three tablets per day and physical therapy. 

Four years postoperatively, the lower extremity weakness persisted along with the in-toeing gait which was attributed to femoral anteversion. The patient underwent a right subtalar arthrodesis with tendon transfer. She continued to walk with a limp, and her feet were still weak bilaterally, both of which had been present since before the TCR without any improvement. When the patient was 20 years old, EDX studies of the lower extremities were requested. 

On exam, weakness of both lower extremities was noted, primarily of flexion and extension of the toes. There was also weakness of the dorsiflexors of the ankles and plantar flexors. Patellar and Achilles DTRs were absent bilaterally. Pinprick sensation was decreased in the dorsal and plantar aspects of the feet bilaterally. The upper extremities showed normal muscle strength and sensations; deep tendon reflexes could not be elicited.

Electromyography (EMG)/NCV of the legs

No compound muscle action potentials (CMAPs) could be recorded over the extensor digitorum brevis (EDB) or abductor hallucis on stimulation of the peroneal or tibial nerves. No H-reflex could be recorded; the latency of the M wave was prolonged. There were no sensory potentials on stimulation of the plantar, superficial peroneal, or sural nerves bilaterally (Table 1).

**Table 1:** Nerve Conduction Study Findings of the Patient, Her Father, and Her Brother MDL: Motor distal latency (ms); MCV: motor conduction velocity (m/s); Amp: amplitude (measured in mV for motor and uV for sensory); Lat: latency (ms); ND: not done Normal Values Median nerve MDL: < 4.4 ms Median nerve MCV: > 49 m/s Median nerve CMAP amplitude: > 4 mV Median nerve SNAP amplitude: > 25 uV Median nerve sensory latency: < 3.6 ms Ulnar nerve MDL: < 3.5 ms Ulnar nerve MCV: > 49 m/s Ulnar nerve CMAP amplitude: > 2.5 mV Ulnar nerve SNAP amplitude: > 10 uV Ulnar nerve sensory latency: < 3.5 ms Peroneal nerve MDL: < 6.0 ms Peroneal nerve MCV: > 39 m/s Peroneal nerve CMAP amplitude: > 2 mV

	Median Nerve MDL/MCV/Amp	Median Nerve Sensory Lat/Amp	Ulnar Nerve MDL/MCV/Amp	Ulnar Nerve Sensory Lat/Amp	Peroneal Nerve MDL/MCV/Amp	Sural Nerve Lat/Amp
Patient	8.2/17.4/5.1	Absent	7.4/19.1/3.1	Absent	Absent	Absent
Her Father	ND	ND	6.7/30.8/5.1	Absent	8.7/22.6/0.26	ND
Her Brother	8.0/27.3/4.3	6.7/2.7	6.2/23.4/2.8	10.2/11	Absent	ND

Needle EMG demonstrated decreased motor units and increased polyphasics in the tibialis anterior and gastrocnemius bilaterally. The prolonged M latency raised the question of underlying neuropathy, prompting evaluation of upper extremity nerves.

EMG/NCV and US of the arms

**Figure 1:**
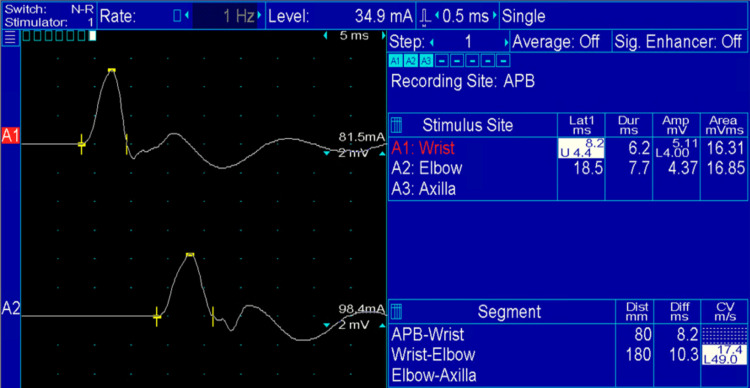
Right Median Nerve Motor Conduction Study Right median nerve motor conduction study:  Median nerve was stimulated at the wrist (top wave) and elbow (lower wave).  Recording was done from the abductor pollicis brevis with surface electrodes.  Note the prolonged distal motor latency (8.2 ms; normal < 4.4 ms) and very low motor conduction velocity (17.4 m/s; normal > 49 m/s). APB: Abductor pollicis brevis

**Figure 2:**
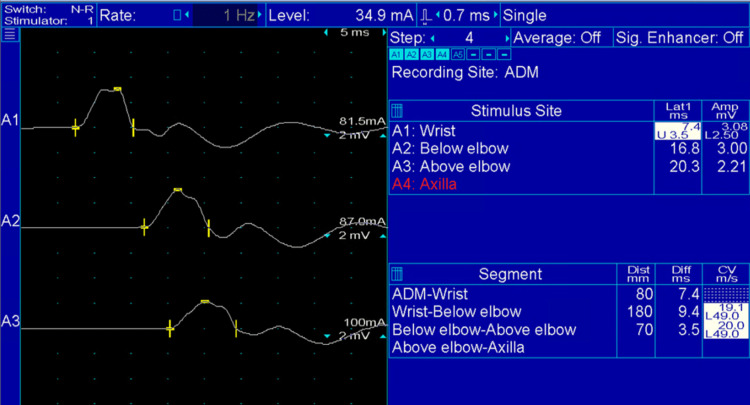
Right Ulnar Nerve Motor Conduction Study Right ulnar nerve motor conduction study: Ulnar nerve was stimulated at the wrist (top wave), below the elbow (middle wave), and above the elbow (lower wave).  Recording was done from the abductor digiti minimi with surface electrodes.  Note the prolonged distal motor latency (7.4 ms; normal: < 3.5 ms) and diffusely slow motor conduction velocity (19.1; normal: > 49 m/s). ADM: Abductor digiti minimi

**Figure 3:**
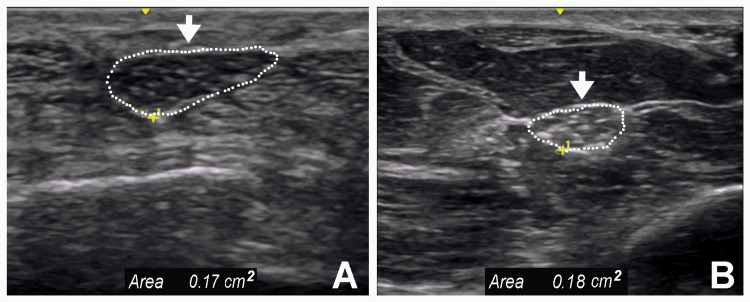
Ultrasound Study of the Right Median Nerve Short axis view of the right median nerve (arrow) showing a uniform increase in its cross-sectional area (A) at the distal wrist crease (17 mm^2^; normal: < 12 mm^2^) and (B) the mid forearm (18 mm^2^; normal < 8 mm^2^)

The median and ulnar nerves showed significant prolongation of distal motor latency and marked slowing conduction velocity (Figures 1-2; Table 1). The sensory nerve action potentials were absent. An US study showed a diffuse increase in the CSA of the median nerves (Figure 3A and Figure 3B).

 

The abnormal EDX study (significant diffuse slowing of conduction without temporal dispersion) suggested a demyelinating polyneuropathy (with additional axonal involvement in the lower extremity nerves), most likely CMT disease. Genetic studies subsequently revealed a PMP22 mutation, confirming CMT1A disease.

Family history and their electrodiagnostic studies

Following the patient’s diagnosis of CMT1A disease, several members of her family underwent EDX studies to evaluate for CMT disease. The patient’s 46-year-old father reported a several-year duration of gait abnormalities. He had high-arched feet bilaterally and restless leg syndrome, as well as muscle cramping of the upper and lower extremities and the chest wall. On exam, there was weakness of the dorsiflexors and plantar flexors of the feet bilaterally and the extensors and flexors of the toes. The right median and peroneal nerves showed prolongation of distal motor latency with a significant decrease in motor conduction velocity in the forearm segment (Table 1). The amplitude of CMAPs over the EDB was decreased. No sensory potential could be recorded on stimulation of the right ulnar nerve. The findings of significant slowing of motor conduction and absent sensory potentials supported the diagnosis of CMT1A disease.

The patient’s 22-year-old brother reported a history of weakness of the feet bilaterally, high-arched feet, restless leg syndrome, muscle cramping, and unstable ankle joints. On exam, there was significant weakness of the dorsiflexors, plantar flexors, and evertors of the feet as well as extensors and flexors of the toes. There was also wasting and weakness of the abductor pollicis brevis bilaterally. The right median and ulnar nerves showed significant prolongation of distal motor latency and decreased motor conduction velocity in the forearm segment (Table 1). The right peroneal nerve stimulation did not evoke CMAPs over the EDB, and no sensory potentials could be recorded on stimulation of the right superficial radial nerve. The findings were consistent with the clinical diagnosis of CMT1A disease. 

## Discussion

To our knowledge, only two cases have been reported in the literature which discuss the relationship between CMT disease and TCS [12,13]. In both of these cases, the patients were initially diagnosed with CMT disease until a lumbar MRI confirmed spinal cord tethering. Gavanozi and colleagues described a 22-year-old man with a six-month history of gait instability, painful distal dysesthesia in the legs, and neurogenic bladder [13]. He also had a 10-year history of progressive intrinsic foot muscle weakness and atrophy bilaterally. CMT disease was initially considered. The patient performed 4-5 self-catheterizations daily. On exam, distal leg muscle weakness bilaterally, decreased DTRs in the legs, and hypoesthesia of the anterior and lateral right leg were noted. The motor and sensory nerve conduction findings were normal. Family history was negative for CMT disease although positive for neurofibromatosis type I. A lumbar MRI revealed a tethered cord, and he underwent a TCR with improved bladder function and stabilization of his symptoms. These authors suggested considering TCS in young patients with foot deformities. 

Souza and colleagues reported a 23-year-old man with progressive difficulty with walking since childhood as well as a three-year history of urinary retention [12]. Pes cavus, hammer toes, and peroneal atrophy were observed on exam. Electroneuromyography revealed chronic denervation with an axonal pattern. CMT disease was suspected as the diagnosis. A subsequent lumbar MRI demonstrated a lipomeningocele at the lumbosacral region with spinal cord tethering. A TCR was performed with immediate improvement of symptoms. These authors described that pes cavus and hammer toes may strongly resemble CMT disease which obfuscates the diagnosis of TCS. However, they acknowledged that urinary symptoms are classic findings in cauda equina syndrome and not neuropathy. In both the previous cases, the patients lacked midline cutaneous stigmata in the lumbosacral area which are commonly found in TCS. Additionally, neither of these patients underwent EDX or US studies. 

The present case highlights the diagnostic challenge due to the common features of both CMT disease and TCS (Table 2).

**Table 2:** Differentiating Features Between Charcot-Marie-Tooth Type 1A Disease and Tethered Cord Syndrome MRI: Magnetic resonance imaging

Differentiating Features	Charcot-Marie-Tooth Disease Type 1A	Tethered Cord Syndrome
Signs and Symptoms	Distal muscle weakness, sensory loss, decreased deep tendon reflexes, and skeletal deformities (pes cavus)	Cutaneous stigmata (nevi, lipomas, hair tufts, dermal sinuses, hemangiomas), progressive lower extremity orthopedic deformities, bowel and bladder deficits (neurogenic bladder with repeated urinary tract infections and urinary incontinence), progressive sensorimotor deficits, gait abnormalities (toe-walking and clumsiness), easy leg fatiguability, and scoliosis
Imaging	High-resolution ultrasound: diffusely increased cross-sectional area of nerves	Lumbar MRI: low-lying conus medullaris (below the L1-2 disc space) and thickened filum terminale (diameter > 2 mm)
Neurophysiology	Slowing of nerve conduction velocities (ulnar and median motor nerve conduction velocity ≤ 38 m/s)	Urodynamic testing: detrusor hyperreflexia common
Pathology	Nerve biopsy: well-formed onion-bulb formations on histopathology	

Unlike the two previously reported cases involving CMT disease and TCS where the patients were initially misdiagnosed with CMT disease and later with TCS after lumbar imaging, our patient was diagnosed clinically with TCS although she had a negative lumbar MRI, normal UDS, and normal renal sonogram. Following a TCR, she was subsequently diagnosed with CMT disease by EDX studies with genetic confirmation. We propose that the diagnosis of TCS may have been presumptive. The bowel abnormalities resolved two years prior to the TCR, although she continued to experience nocturnal enuresis. While the patient attained improvement in the low back pain after the TCR, the gait abnormalities and weakness of the feet persisted without any relief. Both of these findings may have been related to CMT disease instead of TCS. While bladder control problems are much more likely in TCS, those have been reported in patients with CMT [14].

This case underscores the importance of performing EDX studies in the upper extremities, as the muscle atrophy in the lower extremities may preclude adequate evaluation of motor conduction. The absence of SNAPs in the lower extremities favors polyneuropathy but is not conclusive as it has been reported in TCS [15]. In this patient, one clue was the prolonged M latency which raised the suspicion of CMT leading to the upper extremity EDX studies. While the patient’s right leg pain and paresthesia may be due to lumbosacral radiculopathy related to the previously operated tethered cord, the neuropathic pain is more likely related to CMT disease. Performing thorough EDX studies and collecting a comprehensive family history prior to the TCR with particular attention to gait and skeletal deficits would most likely have confirmed CMT disease, thereby making the need for a TCR questionable. 

The role of US in the evaluation of patients presenting with overlapping signs and symptoms of CMT and TCS is promising. Given the difficulty in obtaining reliable diagnostic information by EDX studies of the lower extremities in such patients, an US study of the tibial, peroneal, and sural nerves documenting a uniform increase in CSA has the potential for rapid differentiation of CMT from TCS. 

It is not uncommon for patients to present clinically with signs and symptoms of TCS yet have a normal-lying CM and normal FT thickness [7,8]. It has been suggested that the radiographic criteria for TCS alone are inadequate for the diagnosis of TCS and that the clinical presentation is imperative to confirm the diagnosis [7]. The benefit of a TCR is also controversial in patients with normal lumbar imaging [10]. However, it has been reported that pain and bowel/bladder incontinence are often improved after TCR in patients without radiological evidence of a tethered spinal cord [8]. 

## Conclusions

Due to the overlapping signs and symptoms of CMT disease and TCS, both these conditions should be considered in the differential diagnosis of the other. EDX studies are pivotal in the diagnostic evaluation and should include upper extremity studies also as findings may be non-diagnostic in the lower extremities. EDX and US testing is important prior to a TCR, particularly in cases when a lumbar MRI is negative and when patients have a family history of pes cavus. A comprehensive family history is warranted before a TCR is performed, with special attention to members who may have skeletal or gait abnormalities which may uncover an unsuspecting diagnosis of CMT disease. 
